# Construction of competing endogenous RNA network and identification of novel molecular biomarkers in colon cancer: A bioinformatic analysis

**DOI:** 10.1097/MD.0000000000025369

**Published:** 2021-04-09

**Authors:** Gu Xi, Xu Ziyu, Liu Yiting, Liu Zonghang, Zheng Lifeng

**Affiliations:** a Department of General Surgery, The Nanjing Jiangbei People's Hospital, Nanjing; b Department of Imaging, Medical College of Nantong University, Nantong, Jiangsu, China.

**Keywords:** bioinformatic analysis, colon cancer, competing endogenous RNA network, long noncoding RNAs

## Abstract

Colon cancer patients suffer from high incidence and mortality rates worldwide. More novel molecular biomarkers should be used for the diagnosis and treatment of colon cancer. Long noncoding RNAs (lncRNAs) are found to be involved in colon cancer tumorigenesis and metastasis. This study aimed to identify novel lncRNAs in colon cancer.

Two independent datasets (GSE70880 and GSE110715) were downloaded from the Gene Expression Omnibus database and merged with the sva package. R software was used to distinguish differentially expressed lncRNAs and mRNAs in the merged dataset. The competing endogenous RNA (ceRNA) network was constructed using differentially expressed lncRNAs and mRNAs with Cytoscape. Differentially expressed RNAs in the ceRNA network were further verified using the Cancer Genome Atlas database. Gene oncology analysis, Kyoto Encyclopedia of Genes and Genomes pathway enrichment and survival analysis were also performed to identify hub genes.

A total of 99 differentially expressed lncRNAs and 95 differentially expressed mRNAs were identified in the merged database. Ten lncRNAs, 8 miRNAs, and 6 mRNAs were involved in the ceRNA network, in which LINC00114 and UCA1 were highly expressed in colon cancer. They were both associated with early tumor stages and might be used for the early diagnosis of colon cancer. High expression of LINC00114 can lead to poor overall survival of colon cancer patients. Furthermore, new pathways such as LINC00114/miR-107/PCKS5, UCA1/miR-107/PCKS5, and UCA1/miR-129-5p/SEMA6A were identified.

Two novel lncRNAs (LINC00114 and UCA1) in colon cancer were identified by bioinformatics analysis. They might contribute to the occurrence and development of colon cancer. In addition, LINC00114 may be involved in the overall survival of colon cancer patients.

## 1 Introduction

Colon cancer is one of the most common malignant gastrointestinal tumors. Worldwide the incidence of colon cancer was in the third place lower than lung and breast cancer. The mortality rate of colon cancer was in the second place lower than that of lung cancer.^[1]^ The incidence (11.13%) and mortality (8.15%) rate of colon cancer were both in fifth place in China.^[2]^ Colon cancer patients are divided into different stages according to the tumor/node/metastases system. Most patients are found in advanced stages because of their asymptomatic nature and usually have a poor prognosis.^[3]^ Although molecular biomarkers such as carcinoembryonic antigen (CEA) and carbohydrate 19-9 (CA19.9) are associated with early diagnosis and prognostic assessment of colon cancer, they are not currently used in clinical practice as they are unreliable.^[4]^ Therefore, novel biomarkers need to be identified and applied to the diagnosis or treatment of colon cancer.

Researchers have focused on identifying DNA, RNA, or proteins as molecular biomarkers to detect cancers. Recently, studies have investigated the regulation of non-coding RNAs, which may have an impact on development of cancers.^[5]^ LncRNAs, which contain more than 200 nucleotides belong to noncoding RNA families. They have no protein-coding ability and were once considered to be transcriptional noise.^[6,7]^ The aberrant expression of lncRNAs in cancers has been demonstrated to influence apoptosis, proliferation, transfer, and migration of cancer cells by regulating DNA, RNA, protein expression and interaction between them.^[8]^ In addition, mounting evidence indicates that lncRNAs are implicated in a variety of cancer biological processes, including chromatin interaction, transcription regulation, and so on.^[9,10]^ Generally, lncRNAs exert their function through regulating underlying target genes expression at the epigenetic, transcriptional, and posttranscriptional levels.^[11]^ LncRNAs can competitively bind with miRNA to regulate mRNA expression which is considered as ceRNA theory. CeRNA plays an important role in the occurrence and development of cancer.^[12]^ Qiong Wu et al found that lncRNA MALAT1 regulated HMGB1 expression by sponging miR-129-5p to induce colon cancer development.^[13]^ Changwei Lin et al found that LINC01234 upregulated SHMT2 expression by competitively binding to miR-642a-5p to promote colon cancer proliferation.^[14]^

GSE70880 and GSE110715 were downloaded from the Gene Expression Omnibus (GEO) database in our study. After gene reannotation and batch normalization, we performed gene expression analysis to identify differentially expressed genes (DEGs). Then the ceRNA network was constructed using differentially expressed lncRNAs and mRNAs. The results were validated using the Cancer Genome Atlas (TCGA) database. Eventually we find that LINC00114 and UCA1 are upregulated in colon cancer tissues compared to normal tissues. High expression of LINC00114 leads to improved overall survival (OS) in colon cancer patients. The expression of LINC00114 is higher in stage I and UCA1 is lower, indicating that they can be used for early colon cancer diagnosis. In addition, LINC00114/miR-107/PCSK5, UCA1/miR-107/PCKS5, and UCA1/miR-129-5p/SEMA6A axes may play an important role in colon cancer carcinogenesis and require further research.

## 2 Materials and methods

### 2.1 Gene expression microarray datasets

We downloaded the RNA expression profiles from the GEO database. Datasets that met these criteria were considered eligible:

1. Studies focused on colon cancer patients;
2. The technology and platform information were available for studies;
3. Studies contained colon cancer tissues and adjacent non-tumorous normal tissues.

Two datasets GSE70880 (including 20 pairs of colon cancer samples and adjacent non-tumor samples from platform GPL19748) and GSE110715 (including 6 pairs of colon cancer samples and adjacent non-tumor samples from platform GPL18180) were included for further study. The details of the two datasets are shown in Table 1.

**Table 1:** Details of datasets and merged dataset from the GEO database.

Series accession	Platform	Tumor samples	Normal samples	Total differentially expressed genes	Differentially expressed lncRNA	Differentially expressed mRNA
GSE70880	GPL19748	20	20	884 (Up:379; Down:505)	113 (Up:61; Down:52)	771 (Up:318; Down:453)
GSE110715	GPL18180	6	6	498 (Up:199; Down:299)	277 (Up:108; Down:169)	221 (Up:91; Down:130)
Merged	–	26	26	194 (Up:83; Down:111)	99 (Up:53; Down:46)	95 (Up:30; Down:65)

GEO = Gene Expression Omnibus.

### 2.2 Differentially expressed genes

We merged two datasets into one that included 26 pairs of colon cancer samples and adjacent non-tumor samples. To eliminate bias between the two datasets, we used sva packages with R software (version 3.6.2) to batch-normalize the merged dataset. We then used the limma package to identify DEGs. Adjusted *P*-value < .05 and log | fold change (FC) | > 1 were the selection criteria. DEGs that met the criteria were considered statistically significant. The results were visualized by volcano plots and heatmaps using R software.

### 2.3 Construction of ceRNA network

We used the miRcode database (https://www.mircode.org) to predict miRNAs targeted by differentially expressed lncRNAs. MiRDB (https://www.mirdb.org), TargetScan (https://www.targetscan.org) and miRTarBase (https://www.tarbase.com) were used to predict differentially expressed mRNAs interacting with miRNAs. Only all three databases predicted mRNAs were included in the lncRNA-miRNA-mRNA network. The network was visualized using the Cytoscape software.

### 2.4 Functional enrichment analysis and protein-protein interaction network

Webgestale (https://www.webgestalt.org) were used to perform Gene Oncology (Go) analysis. The functions of Go analysis were divided into biological processes (BP), molecular functions (MF) and cellular component (CC). Pathway analysis was performed using the Kyoto Encyclopedia of Genes and Genomes (KEGG) pathway enrichment. A protein–protein interaction (PPI) network was constructed using STRING (https://string-db.org) online database.

### 2.5 Survival analysis and expression level of lncRNAs and mRNAs in the ceRNA network

We downloaded the RNA expression profiles and clinical information of colon cancer patients from the TCGA database. Details of the clinical information are shown in Table 2. We then performed Kaplan–Meier (KM) analysis using the R survival package based on the expression levels of lncRNAs and mRNAs in the ceRNA network. *P*-value < .05 was considered statistically significant. The expression levels of selected lncRNAs were validated with the TCGA database using Student's *t* test.

**Table 2:** The clinical information of colon cancer patients in TCGA database.

Characteristics	Number of cases	Percentages (%)
Gender		
Female	180	46.75
Male	205	53.25
Age		
>60	273	70.91
≤60	112	29.09
Vital status		
Alive	314	81.56
Dead	71	18.44
Stage		
Stage I	66	17.14
Stage II	151	39.22
Stage III	103	26.75
Stage IV	54	14.03
Unknow	11	3.86
Topography		
T1	9	2.34
T2	68	17.66
T3	263	68.31
T4	44	11.43
Tis	1	0.26
Lymph node stage		
N0	231	60
N1	88	22.86
N2	66	17.14
Metastasis		
M0	286	74.29
M1	54	14.03
Mx	45	11.69

TCGA = the Cancer Genome Atlas.

### 2.6 Ethics and dissemination

The study protocol was approved by the Ethics Committee of Nanjing Jiangbei People's Hospital. All data were downloaded from public databases. No patients were involved in our study and there was no written informed consent.

## 3 Results

### 3.1 Gene expression profile from GEO database

**Figure 1:**
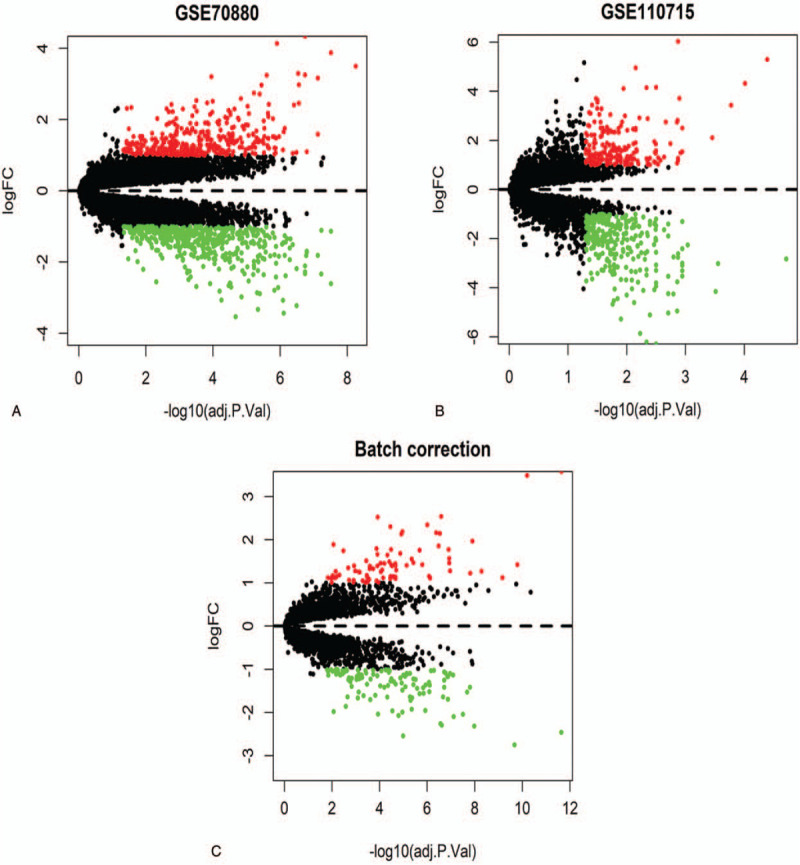
Volcano plot of differentially expressed genes in datasets from GEO database. (A) GSE70880 dataset. (B) GSE110715 dataset. (C) The merged dataset. Green dots indicates downregulated genes, red dots indicates upregulated genes, black dots indicates no significant difference in expression. FC = fold change, GEO = Gene Expression Omnibus.

Two gene expression profiles (GSE70880 and GSE110715) and their platform information were downloaded from the GEO database. There were 505 downregulated genes and 379 upregulated genes in GSE70880 (Fig. 1A). GSE110715 contained 299 downregulated genes and 199 upregulated genes (Fig. 1B). We merged these two datasets into one. After batch normalization, there were 111 downregulated genes and 83 upregulated genes in the merged dataset (Fig. 1C). The names of these DEGs are listed in Table 3. The merged dataset contained 99 differentially expressed lncRNAs (up: 53, down: 46, Fig. 2A) and 95 differentially expressed mRNAs (up: 30, down: 65, Fig. 2B).

**Table 3:** A total of 194 differentially expressed genes are identified from the merged dataset.

lncRNAs	Upregulated	LINC02595,LINC01614,LINC01655,CRNDE,AC009262,TRPM2,AL365226,LINC01186,LINC02418,AL513123,LINC00858,FEZF1,LINC01555,LINC02253,HECW1-IT1,LINC02487,FAM222A,AP000526,LINC01124,PVT1,LINC01594,AC104958,DLEU7,LINC02577,FIRRE,AC007128,LINC02331,LINC02257,AL135999,ARNTL2,UCA1,LINC00114,DUXAP8,MNX1,ZFAS1,LINC02263,CRAT37,CASC19,AL365181,AC099792,AL136987,AP000542,MIR44352HG,AC007405,CYTOR,MAP3K20,ELFN1,LUCAT1,AC100861,ISM1,LINC00920,LINC01235
	Downregulated	AF064860,DIRC3,AC104117,SLC25A21,AL391704,AC104260,TARID,NKAIN3IT1,AF165147,HAGLR,AC017015,AC026391,PCAT18,AL158211,B3GALT5,LINC02023,AL391807,AL591501,AC017067,ADAMTS9,CDKN2B,LINC02024,CARMN,GDNF,AL442638,AC073862,MIR4307HG,AL136317,LINC00702,CYP1B1,LINC01474,FENDRR,PWRN1,DPP10,AL024497,AL590302,AC006007,ADAMTS9,AC022034,AC100826,MAMDC2,AC007182,PGM5,HAND2,AC087463,AL136369
mRNAs	Upregulated	CEMIP,MMP1,WNT2,LEMD1,CHI3L1,SLC7A11,NKD2,INHBA,GJB3,MTHFD1L,TLX1,NEBL,HCAR3,NOX4,HSPH1,TRPM2,COL8A1,MUC5AC,ADAMTS2,CCNB1,GRIN2B,AKR1C8P,HECW2,MAP3K20,SIX1,TM4SF19,RNF43,SGIP1,KCNJ15,EDNRA
	Downregulated	BLK,UNC5C,SLC22A18AS,DDR2,SEMA6A,ADIPOQ,ST6GALNAC6,PLP1,HSD17B2,MYH11,KRT222,PPARGC1B,CDON,TENM1,AC020907,HDAC9,TMEM72,SLC9A9,PAPPA2,KRTAP222,TMEFF2,TTLL7,DPF3,RAB9B,REP15,PDE7B,ANK2,AC124312,SLC16A9,LRRC3B,LPP,CSRP1,SLIT3,PPARGC1A,ADCYAP1R1,DCLK1,NOVA1,SPEG,P2RY1,FBXO32,DISP2,RIC3,EDIL3,PCSK5,ROR1,MYOCD,LMO3,STMN2,LGI1,FOXP2,GSTM3,JCHAIN,TNXB,B3GALT5,CFL2,UGT2A3,LMOD1,RNF150,INSL5,VSTM2A,MAL,PTCHD1,XKR4,TPH1,LYVE1

**Figure 2:**
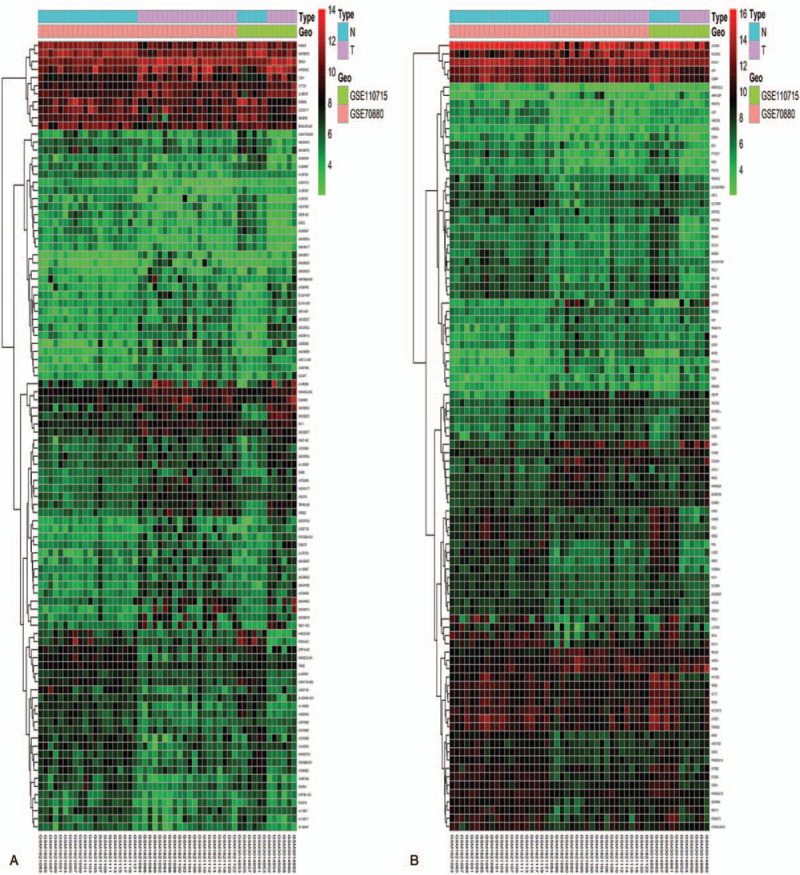
Heatmap of differentially expressed lncRNAs and mRNAs in the merged dataset (26 colon cancer samples and 26 non-tumor samples). (A) Heatmap of differentially expressed lncRNAs. (B) Heatmap of differentially expressed mRNAs. The green color indicates downregulated genes; the red color indicates upregulated genes; the black color indicates no significant difference in expression.

### 3.2 CeRNA network

**Figure 3:**
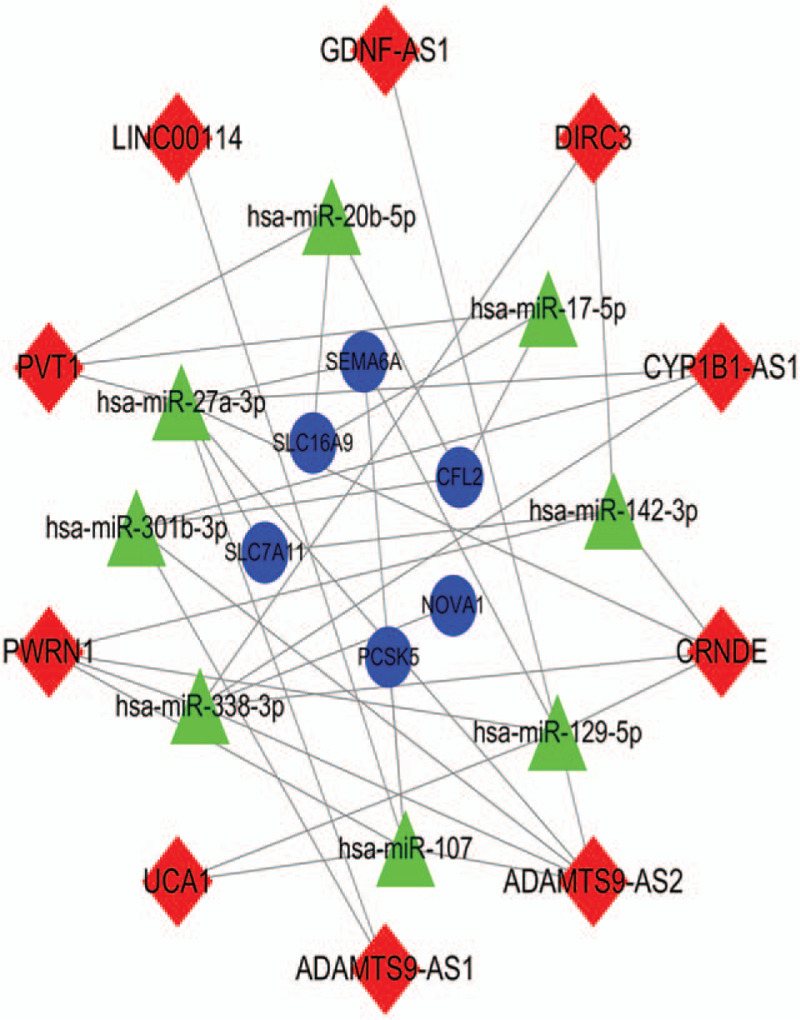
CeRNA network. Red diamond indicates lncRNAs. Green triangle indicates miRNAs and blue circle indicates mRNAs. ceRNA = competing endogenous RNA.

Ten lncRNAs, eight miRNAs, and six mRNAs were involved in the ceRNA network (Fig. 3). There were 24 nodes and 39 lines in the ceRNA network. Six miRNAs interact with lncRNA ADAMTS9-AS1, indicating that it might play an important role in the ceRNA network.

### 3.3 Go terms and KEGG pathway analysis and PPI

**Figure 4:**
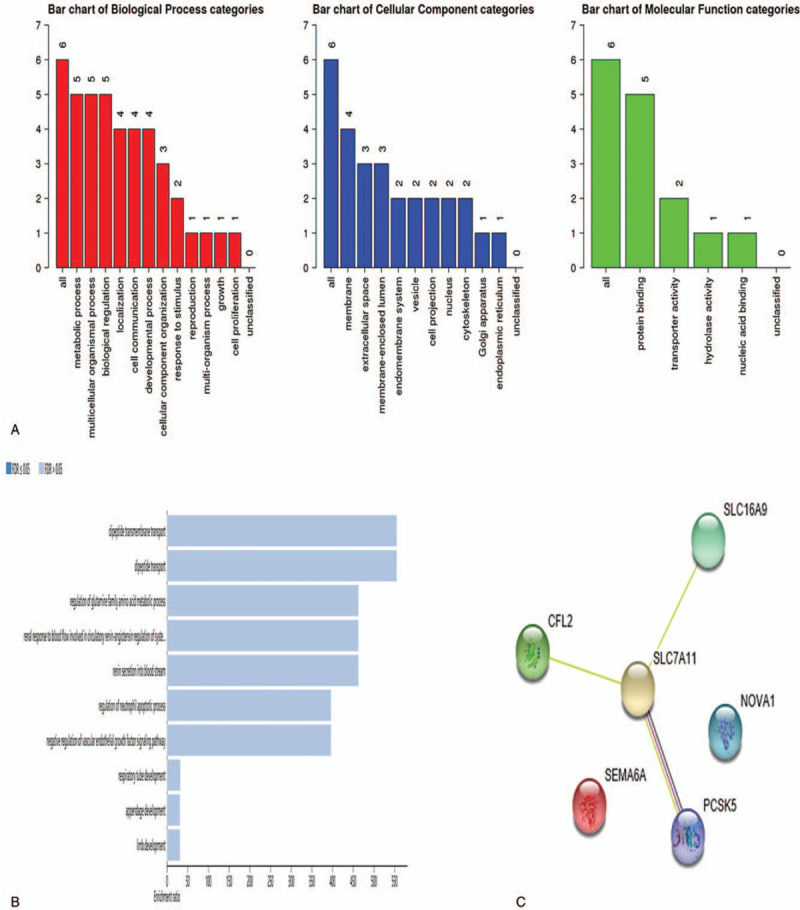
Functional analysis of mRNAs in ceRNA network. (A) Go analysis of mRNAs. (B) KEGG pathway analysis of mRNAs. (C) PPI network of mRNAs. ceRNA = competing endogenous RNA, Go = Gene ontology, KEGG = Kyoto Encyclopedia of Genes and Genomes, PPI = protein–protein interaction.

We performed GO terms and KEGG pathway analysis to determine the potential functions of mRNAs included in the ceRNA network. We found that biological processes were associated with metabolic processes, multicellular organismal processes and biological regulation. Cellular components gathered in the membrane, extracellular space and membrane-enclosed lumen. Molecular functions were associated with protein binding. All these results are shown in Figure 4A. KEGG pathway analysis showed that mRNAs were mainly involved in dipeptide transmembrane transport and dipeptide transport (Fig. 4B). However, the results were not statistically significant, and the false discovery rate (FDR) > 0.05. PPI results showed that SLC7A11 had the highest connectivity and three mRNAs interacted with it (Fig. 4C).

### 3.4 Survival analysis

**Figure 5:**
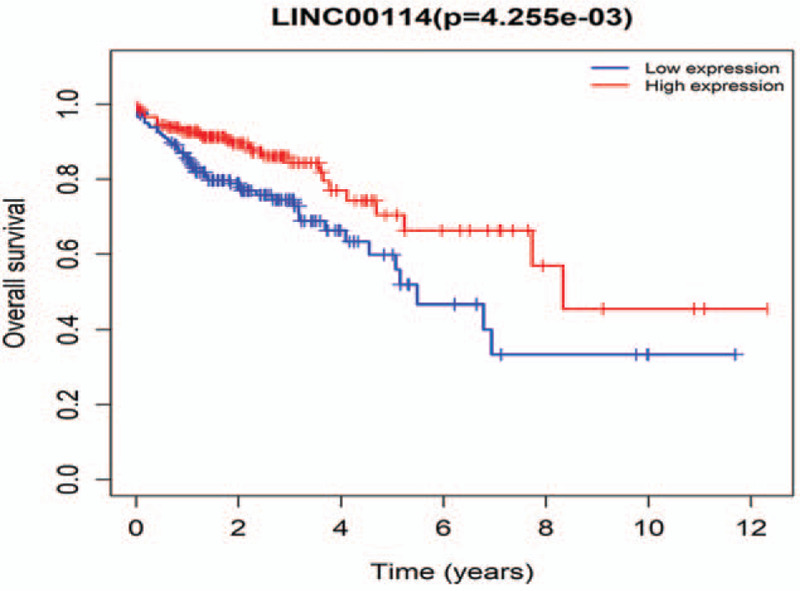
KM analysis result of LINC00114. KM = Kaplan–Meier.

**Figure 6:**
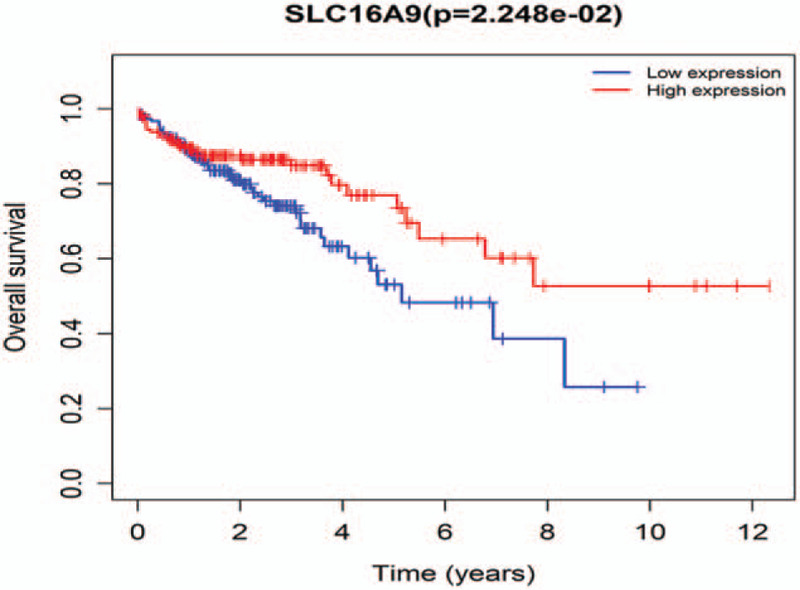
KM analysis result of SLC16A9. KM = Kaplan–Meier.

Gene expression profiles and clinical information of 437 samples (398 tumors and 39 adjacent normal tissues) of colon cancer patients were obtained from the TCGA database. Nineteen tumor samples were deleted because of a lack of follow-up time. Eventually, 379 colon tumor samples were included in the analysis. In order to identify prognostic genes of colon cancer patients in the ceRNA network, we performed survival analysis based on gene expression and survival time of patients in the TCGA database. We find that LINC00114 is associated with OS in colon cancer patients (*P* = 4.255e−03, Fig. 5). Colon cancer patients with high LINC00114 expression have longer survival time than those with low expression. In mRNAs, SLC16A9 is found to be associated with OS in colon cancer patients (*P* = 2.248e−02, Fig. 6). High expression of SLC16A9 leads to improved OS in colon cancer patients.

### 3.5 LncRNAs expression levels and clinicopathological variables

**Figure 7:**
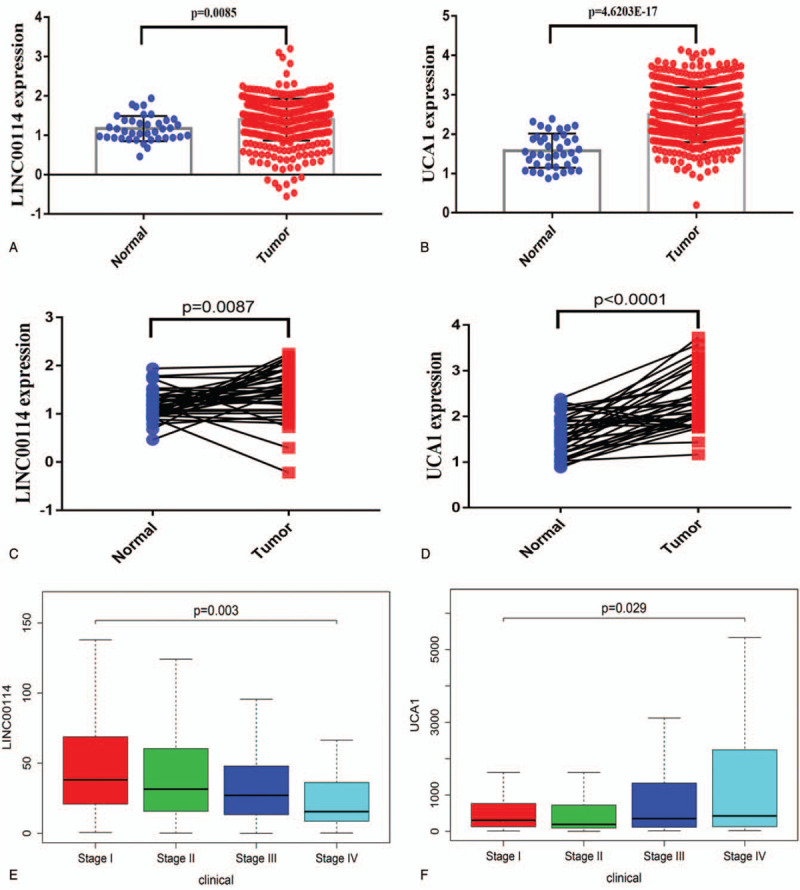
LnRNAs expression levels. (A and B) The expression level of LINC00114 and UCA1 between tumors and normal tissues using TCGA database. (C and D) The expression level of LINC00114 and UCA1 in paired colon cancer tissues using TCGA database. (E and F) The correlations between LINC00114, UCA1 expression levels and clinicopathological variables. TCGA = the Cancer Genome Atlas.

The expression levels of LINC00114 and UCA1 were validated using the TCGA database. LINC00114 and UCA1 are both highly expressed in colon cancer tissues compared to normal tissues in the TCGA database (Fig. 7A and B). The results are consistent when using paired colon cancer tissues from the TCGA database (Fig. 7C and D). We then investigated the correlation between lncRNAs expression levels and clinicopathological characteristics and find that the expression of LINC00114 decreased with increasing tumor stage (*P* = .003, Fig. 7E). However, the relationship between UCA1 expression and tumor stage is the opposite (*P* = .029, Fig. 7F). These results indicates that both LINC00114 and UCA1 can distinguish colon cancer patients from different stages. In conclusion, LINC00114 has the potential to be a prognostic biomarker and they can be used for early diagnosis of colon cancer.

## 4 Discussion

In 2011, the ceRNA theory was proposed by Salmena et al. They found that miRNAs could target the same miRNA response elements (MREs) in different RNAs. LncRNA and mRNA sharing the same MRE competed with each other for limited miRNA and mRNA expression was depressed.^[12]^ LncRNAs act as ceRNA that play an important role in cellular biological processes and tumorigenesis.^[15]^ The ceRNA network has been constructed in different types of cancers including gastric cancer,^[16]^ tongue squamous cell carcinoma,^[17]^ hepatocellular carcinoma,^[18]^ breast cancer,^[19]^ and so on. In 2018, Zhang et al constructed a lncRNA-associated ceRNA network in colon cancer using the TCGA database to identify biomarkers. Finally, they constructed a ceRNA network including 64 lncRNAs, 18 miRNAs, and 42 mRNAs and found that mRNAs were enriched in many cancer-related pathways.^[20]^

In our study, we constructed a ceRNA network using the GEO database and verified it in the TCGA database. We included 10 lncRNAs, 8 miRNAs, and 6 mRNAs in the ceRNA network. Among these lncRNAs, LINC00114 is confirmed to be associated with improved OS in colon cancer patients. LINC00114 and UCA1 are confirmed to be associated with the early stages of colon cancer. Go analysis reveals that mRNAs were associated with many cancer-related processes.

Few studies have focused on LINC00114 which mapped on chr21. Lv et al found that LINC00114 is highly expressed in colorectal cancer. It can bind to EZH2 and DNMT1 to induce promoter methylation of miR-133b and miR-133b expression was downregulated. It also sponges miR-133b to regulate NUP214 expression.^[21]^ Han et al demonstrated that LINC00114 plays an important role in nasopharyngeal carcinoma (NPC). It was upregulated in NPC and could be used as a diagnosis marker. It competitively sponges miR-203 to regulate the ERK/JNK pathway to promote the progression and radioresistance of NPC.^[22]^ Our study predicts that LINC00114 is upregulated in colon cancer tissues, which is consistent with Lv's study. It may regulates PCSK5 expression by binding to miR-107. The LINC00114/miR-107/PCKS5 axis may plays an important role in colon cancer tumorigenesis and requires further research.

LncRNA UCA1 which mapped on chr19 has been demonstrated involved in many different tumors such as non-small cell lung cancer,^[23]^ renal cell carcinoma,^[24]^ prostate cancer,^[25]^ and so on. Few studies have focused on the function of UCA1 in colon cancer. Cui et al find that UCA1 promotes colon cancer progression by binding to miR-28-5p and regulates HOXB3 expression.^[26]^ Cao et al find that UCA1 inhibits the carcinogenesis and metastasis of colon cancer by regulating the miR-185-5p/MAPK14/MAPKAPK2/HSP27 axis.^[27]^ In our study, we find that UCA1 expression is upregulated in colon cancer and is associated with early tumor stage. We also predict two novel pathways, UCA1/miR-107/PCKS5 and UCA1/miR-129-5p/SEMA6A in colon cancer.

SLC16A9, also called monocarboxylate transporter 9 (MCT9) is a plasma membrane transporter belonging to the monocarboxylic acid transporter family SLC16. SLC16A9 is responsible for carnitine and creatine, which is presumably transported at the basolateral membrane of enterocytes.^[28]^ SLAC16A9 has also been reported to be associated with some cancer types. Fernandez-Ranvier et al found that SLAC16A9 could be a good diagnostic target for distinguishing benign from malignant adrenocortical tumors.^[29]^ Lim et al found that it was highly expressed in diffuse large B-cell lymphoma.^[30]^ What's more, an informatic analysis have found SLC16A9 had potential diagnostic value for indicating the occurrence of colorectal cancer.^[31]^ In our study, we find that SLC16A9 is highly expressed in colon cancer which leads to improved OS. More related molecular experiments should be performed to explore the role of SLC16A9 in colon cancer.

However, there are some limitations in our study. First, although we batch-normalized the datasets, the bias might still exist because they come from two different platforms. Secondly, we only included two datasets and there were 26 pairs of samples. More samples need to be included to obtain more precise results. Last but not the least, there were only six mRNA in ceRNA network which led to poor result of KEGG analysis.

In conclusion, we have constructed a ceRNA network in colon cancer using the GEO database and verified it in the TCGA database. We find that LINC00114 and UCA1 are upregulated in colon cancer which to be associated with the early tumor stage. They have the potential to serve as new biomarkers for colon cancer.

## Author contributions

**Conceptualization:** Gu Xi.

**Data curation:** Xu Ziyu.

**Investigation:** Liu Yiting.

**Project administration:** Zheng Lifeng.

**Writing – original draft:** Gu Xi.

**Writing – review & editing:** Liu Zonghang.
